# Effects of fire timing and snow cover on tallgrass prairie plant re‐emergence phenology, growth rate, and flowering

**DOI:** 10.1002/eap.70213

**Published:** 2026-04-09

**Authors:** Michelle A. Homann, Ellen I. Damschen

**Affiliations:** ^1^ Department of Integrative Biology University of Wisconsin–Madison Madison Wisconsin USA

**Keywords:** community assembly, fire season, grassland management, growing season, prescribed fire, re‐emergence phenology, winter climate change

## Abstract

In a time of unprecedented global change, understanding plant community responses to contemporary disturbance regimes is necessary to improve the predictability of restoration outcomes. Although fires in tallgrass prairies historically occurred throughout the growing season, contemporary prescribed fires are often conducted during the dormant season, in either spring or fall. Dormant‐season burns remove vegetation and litter at different times of year, which has subsequent effects on microclimate dynamics. These dynamics may compound with projected changes in winter climate, including decreased snow cover and more variable soil temperatures. Short‐term responses to microclimate conditions are most detectable early in the growing season, yet plant community metrics are often measured broadly at one or a few points during the peak growing season. Understanding how disturbance timing and a changing climate influence plant community responses through and beyond these early stages of growth is an imperative step toward improving the ability to predict long‐term plant community responses during restoration. To evaluate responses to disturbance and winter climate, we manipulated fire application, fire timing, and snow depth in a tallgrass prairie restoration from 2016 to 2023, then evaluated re‐emergence timing in spring and subsequent effects on relative growth rate and flowering effort throughout the 2023 growing season. Plants re‐emerged earlier and grew more slowly in fall burn treatments than in spring burn and unburned treatments. Within their respective disturbance treatments, plants that re‐emerged faster tended to grow faster, and relative growth rate was positively correlated with the probability of flowering. Winter snow cover was not correlated with re‐emergence or growth rate, but snow removal tended to decrease the probability of flowering. Our results suggest resilience to winter climate change in the short term and demonstrate cascading effects of fire application and timing on tallgrass prairie plant phenology and fitness responses.

## INTRODUCTION

Disturbance has long been understood to be an important factor in regulating plant community dynamics and succession (White 1979). Anthropogenic influence is altering the disturbance regimes that regulate plant communities (Smith et al., 2009), leading to decreasing biodiversity at multiple scales (Alstad et al. 2016). Understanding the drivers of plant community assembly is an integral but difficult part of predicting plant community change (Kraft & Ackerly 2014). The three stages often highlighted in community assembly are dispersal, establishment, and persistence (Grman et al. 2015; Kraft et al. 2015; Noble & Slatyer 1980). These stages, viewed through the lens of a plant establishing from seed, have been examined within the scope of community assembly theory (Hastings 1980; Keddy 1992; Larson & Funk 2016; Maron et al. 2012). In plant communities that consist primarily of perennial plants, however, re‐emergence of part or all of the existing plant community after disturbance is often more likely than establishment via seed (Moora et al. 2009; Pausas et al. 2018). As such, vegetative regeneration is likely to play a substantial role in perennial species responses to disturbance (Larson & Funk 2016).

**FIGURE 1:**
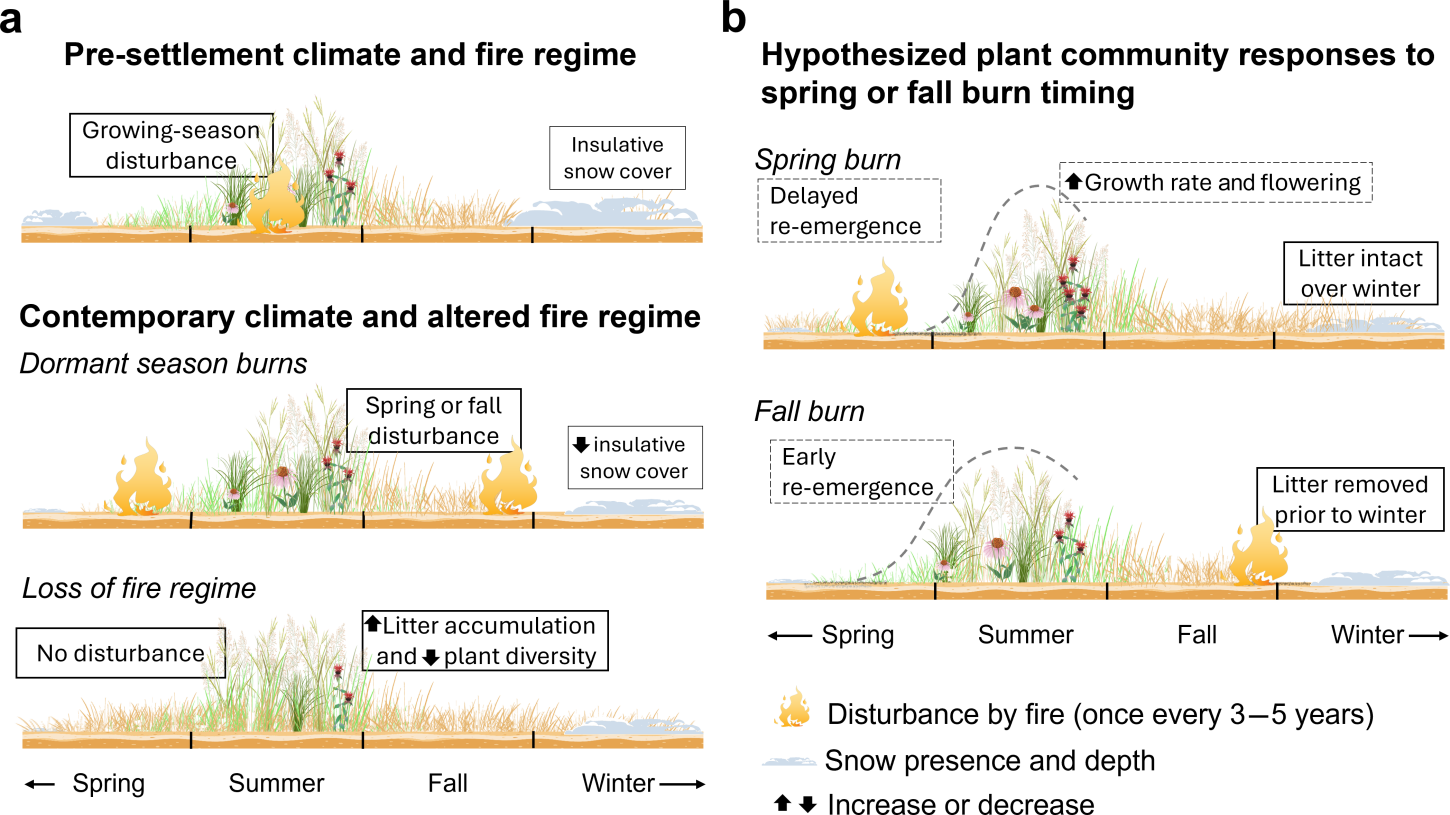
Contemporary winter climate conditions and fire application and timing differ from historical climate conditions and fire regimes (a). These changes alter insulative snow and litter cover over winter through early spring, with possible implications for growing season dynamics. Hypothesized plant community responses to contemporary conditions are shown in dashed boxes (b). Figure created by Michelle Homann using botanical illustrations from Vecta.io.

Understanding how historical disturbance regimes have changed as a result of human impacts is necessary when considering regeneration as a response to disturbance in perennial systems (Brudvig & Catano 2021; Figure 1). From the 1600s to 1900s in the Midwestern United States, grassland fires occurred every 1–5 years during the late summer and early fall (McClain et al., 2021). Contemporary burning, however, is often conducted through prescribed fires during the “dormant season” in late fall (November–December) or spring (March–April) when most native grassland species are not actively growing (Howe 1994; Pavlovic et al. 2011). As many temperate grassland and savanna ecosystems are dominated by fire‐adapted plants that resprout from perennating parts after winter or following disturbance, we expect this relatively recent shift in the timing of fire to have consequences for dormancy breaking and re‐emergence of perennial plants (Russell et al. 2015) as well as implications for restoration outcomes (Copeland et al. 2002; Suding 2001).

Differences in these microclimate conditions following a dormant‐season fire are most prominent at the start of the growing season (Henderson 1982), making re‐emergence phenology one of the most proximal measurable responses to disturbance type and timing. The timing of re‐emergence in spring is governed by species‐dependent cues associated with dormancy break, including soil temperature, soil moisture, and light availability (Anderson et al. 2001; Gillespie & Volaire 2017). Each of these conditions can be altered by the presence or absence of litter (Deutsch et al. 2010; Henn & Damschen 2022; Jensen & Gutekunst 2003). Further, the effects of litter depend on the season(s) in which the litter is present (Carson & Peterson 1990). Fall fires remove insulative litter prior to winter, leading to colder and more variable temperatures over winter while also expediting soil warming in spring (Henn & Damschen 2022). Alternatively, spring fires leave litter intact to insulate the soil surface during the dormant season and early growing season (Deutsch et al. 2010), but there is a trade‐off between the insulative properties of litter and the potential for litter to hinder re‐emergence (Lubbe & Henry 2019; Figure 1).

Although plant community responses to disturbance are often broadly considered through metrics measured during peak growing season, such as diversity or reproductive effort, examining re‐emergence timing as a more proximal response to disturbance could provide important insight into how and why plant communities respond to disturbance. There is evidence that changing spring phenology has effects on plant growth (Zhou et al. 2020, Li et al. 2023). Further, many plants exhibit a trade‐off between cold tolerance—a trait necessary to re‐emerge early in the growing season while freezing is a risk—and growth (Ding et al. 2019). There is also evidence that growth rate and flowering are related in grassland plants (Sun & Frelich 2011). Further elucidating the relationships between re‐emerging, growth, and flowering can improve our understanding of how and why plants respond to disturbance.

During a period of unprecedented global change, it is imperative to examine how factors under some anthropogenic control—such as the timing of prescribed fire—interact with a changing climate to affect plant communities (Wilsey 2021). Winters are changing rapidly in temperate regions, often to a greater extent than growing season conditions (Williams et al. 2015). Climate models predict reduced snow depth and warmer air temperatures in winter for temperate regions (IPCC 2014, pp. 58–62; Kreyling 2010). Perennial grassland species enter dormancy as an adaptation to freezing winter temperatures, and soil temperatures remaining consistently above freezing due to warming winters can break dormancy and cue early re‐emergence (Ott & Hartnett 2012). Plants that break dormancy early may be vulnerable to frost damage, as reduced insulative snow can lead to increased frequency of freeze–thaw events (Henn & Damschen 2022; Henry 2008). Management practices that leave insulative litter intact over winter (e.g., spring burns rather than fall burns) have the potential to reduce or counteract the effects of reduced snow cover (Figure 1). Here we examine how re‐emergence of established perennial species in spring is affected by the application and timing of prescribed fire and winter climate by manipulating fire timing and winter snow depth in a restored tallgrass prairie. We also evaluate whether these re‐emergence patterns have subsequent consequences for vegetative growth and reproductive effort. We hypothesize that: (H1) fall prescribed burns and reduced snow depth will expedite re‐emergence when compared to spring prescribed burns or lack of disturbance and ambient snow depth. We further predict that (H2) a delayed start to the growing season will coincide with increased (H2a) growth rate and (H2b) reproductive effort.

## METHODS

### Study site

This study was conducted at Mounds View Grassland (Iowa County, Wisconsin, USA); a 336‐hectare preserve that is owned and managed by The Prairie Enthusiasts. The study site was restored from agricultural use (corn and soy rotation) to tallgrass prairie via seeding in 2011 and burned in spring every 2–3 years prior to the establishment of eight experimental blocks in September 2016 (Henn & Damschen 2022).

### Experimental design

**FIGURE 2:**
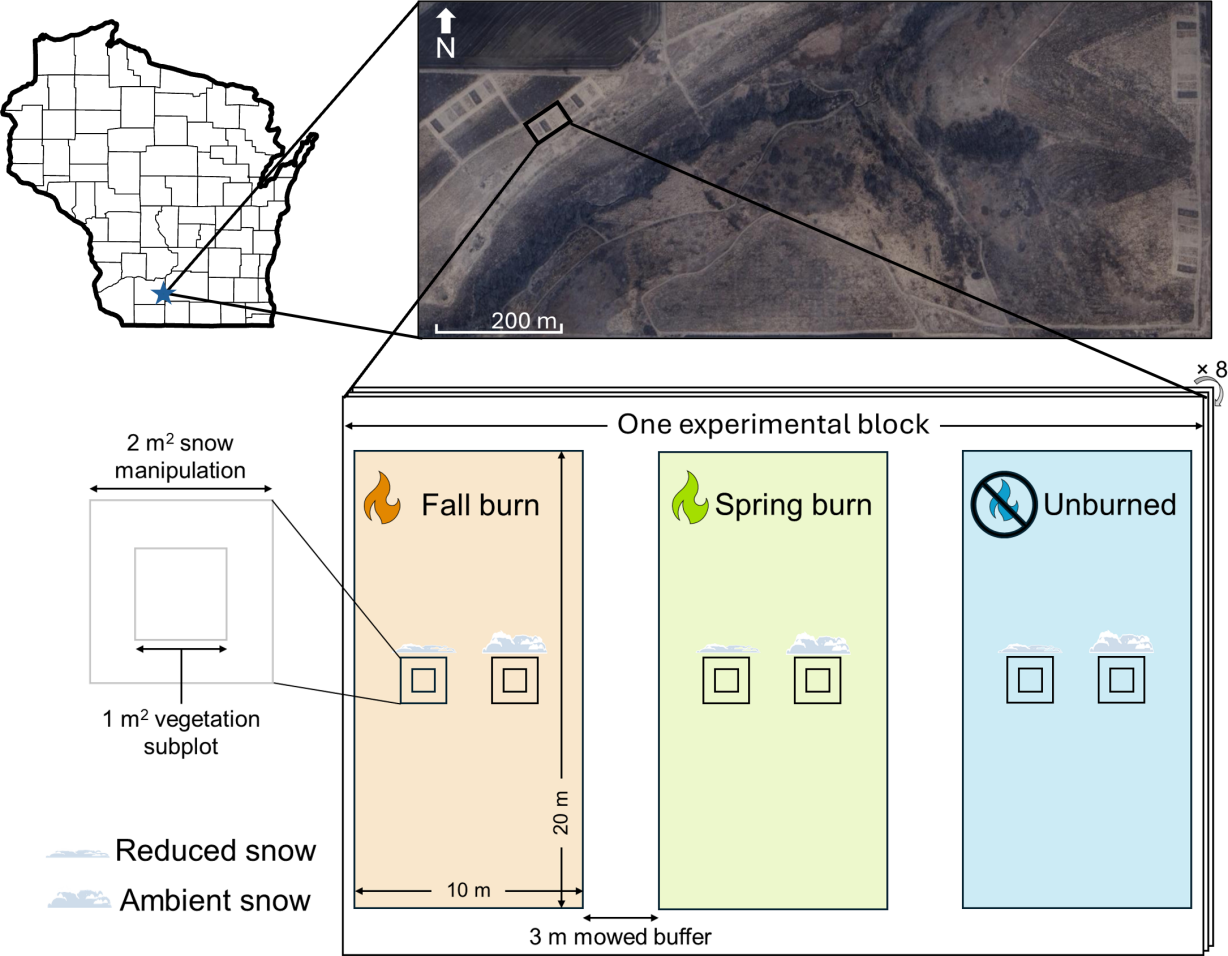
One experimental block at Mounds View Grassland in Iowa County, WI. Colored rectangles represent disturbance treatments and small black squares represent one reduced snow subplot and one ambient snow subplot within each disturbance plot. The pictured experimental design is replicated eight times in a randomized block design. Colors and symbols corresponding to burn treatments here will be used throughout figures. Aerial image in screen capture format, modified from Google Earth (https://www.google.com/earth), retrieved September 20, 2025. Figure created by Michelle Homann using icons of fire from PowerPoint.

To test our hypothesized responses to disturbance and winter snow depth, we applied disturbance treatments in 10 × 20 m treatment plots within each of the eight randomized blocks. We randomly assigned one of three disturbance treatments—fall prescribed burn, spring prescribed burn, and unburned control (hereafter referred to as “unburned”)—to plots within each block, resulting in eight replicate plots for each disturbance treatment (Figure 2). Prescribed burn treatments were applied annually beginning in 2016, with fall burns occurring between November and December and spring burns occurring in April. The fall 2022 and spring 2023 burns prior to this study were conducted on 11 November and 18 April, respectively. A 3‐m buffer strip was mowed between each plot annually in fall to act as a fire break, and burns in each plot were complete, removing the majority of aboveground plant material.

Within each disturbance plot, we established two 2 × 2 m snow manipulation subplots, leaving a 2‐m buffer between each subplot and the disturbance plot edge. We randomly assigned snow manipulations, either ambient (unmanipulated) snow or snow reduction, to each subplot so that each snow manipulation was replicated once in each disturbance treatment plot. There were 48 snow manipulation subplots across the experiment: 24 with ambient snow and 24 with reduced snow. We applied snow reduction treatments any time snow accumulation exceeded 10 cm by manually reducing snow depth to 2 cm with a shovel. Snow manipulations were conducted each year from 2016 to 2018 and 2020 to 2023, and all snowfall events in excess of 10 cm during that timeframe occurred between December and February.

### Species selection

To understand the restored plant community responses to fire application and timing, we chose to measure responses in native species that were well represented across treatments. Four introduced and 22 native tallgrass prairie species compose 90% of the cover and occurrence frequency at our study site. From the 22 native species, we selected 10 forbs and two grasses as focal species in our study (*n* = 12 species; Table 1).

**TABLE 1:** Focal species for re‐emergence, growth, and flowering data collection.

Species	Family	Functional group	Clonality^a^
*Andropogon gerardii*	Poaceae	Grass	Clonal
*Asclepias syriaca*	Asclepiadaceae	Forb	Not clonal
*Echinacea pallida*	Asteraceae	Forb	Not clonal
*Heliopsis helianthoides*	Asteraceae	Forb	Not clonal
*Monarda fistulosa*	Lamiaceae	Forb	Clonal
*Ratibida pinnata*	Asteraceae	Forb	Clonal
*Silphium integrifolium*	Asteraceae	Forb	Clonal
*Solidago canadensis*	Asteraceae	Forb	Clonal
*Solidago rigida*	Asteraceae	Forb	Clonal
*Sorghastrum nutans*	Poaceae	Grass	Clonal
*Symphyotrichum laeve*	Asteraceae	Forb	Clonal
*Tradescantia ohiensis*	Commelinaceae	Forb	Not clonal

^a^ Chadde (2019).

### Data collection

To evaluate plant community composition and re‐emergence dynamics, we estimated percent cover of all species present in 1 × 1 m quadrats centered in each subplot weekly for 2 weeks prior to the spring burn, weekly for 5 weeks after spring burn, every other week from mid‐May through mid‐July, and once more in August for 12 total weeks of sampling. Due to the multilayered canopy of tallgrass prairies, total percent cover in a subplot could exceed 100%.

To determine how disturbance and snow depth influenced the rate of re‐emergence, we marked individual clones of the 12 focal species with sewing pins weekly as they emerged. We collected re‐emergence data prior to the spring burn during the first week of April 2023 and after the spring burn weekly from 21 April through 14 May, biweekly through 13 July, and then once more in mid‐August. We marked at least two individuals per species, if possible, to account for the possibility of losing pins throughout the season. To avoid pseudoreplication, we randomly selected one individual per species per subplot to include in data analyses if more than one individual of a species remained in a subplot by the end of the growing season.

To determine whether growth rate was associated with disturbance, snow treatments, or re‐emergence timing, we measured the tallest ramet or tiller within each marked clone and the widest diameter of each clone in cm during each week of sampling. Before analyses, we multiplied height and diameter for a more comprehensive measure of plant size (in square centimeters; Gibson 2002, p. 181). To determine whether re‐emergence timing had an effect on flowering effort, we quantified the presence or absence of flowers and the number of flowering ramets for each marked individual. We collected flowering data in September and October after target species inflorescences had senesced to minimize damage to developing reproductive structures.

### Data analysis

All analyses were performed in R version 4.2.2 (RStudio Team, 2022), and model assumptions were evaluated using the DHARMa package (Hartig 2022). To determine pairwise differences in the effect of disturbance treatment on each response variable, we conducted post hoc linear contrasts using the “emmeans” and “contrast” functions from the emmeans package in R (Lenth 2023) with a Holm adjustment for multiple comparisons.

To test H1, whether disturbance timing and snow depth influenced re‐emergence phenology, we conducted a time‐to‐event analysis by fitting a Cox proportional hazards model via the coxme package, which allowed us to account for both fixed and random effects (Therneau 2024). We set the ordinal week number in which an individual emerged as the response variable and disturbance treatment and snow treatment as fixed effects in the model. To account for repeated measures at the subplot level within our experimental design, we included a random effect for subplot within plot within block. Additionally, we included a random effect for species, as we expected re‐emergence timing to differ between species but did not have specific predictions regarding how. Finally, we included stratification to ameliorate a violation in the assumption of proportional hazards. We set the stratum for before and after May 14, as that is the point after which total vegetative percent cover no longer differed between burn treatments (*t*(38) = 0.846, *p* = 0.4027; Appendix S1: Figure S1). We did this to limit the influence of early‐season vegetative cover differences on treatment effect estimates later in the season, and including the stratum improved model fit significantly (χ^2^ = 71.255, *p* < 0.001). We also modeled whether disturbance and snow treatments were associated with the total number of individuals that emerged in each treatment. We set the number of individuals as the response variable; disturbance and snow treatments as fixed effects; and block within plot within subplot as a random effect.

To test H2a, whether growth rate was associated with re‐emergence timing or treatments, we used a generalized linear mixed‐effects model via the glmmTMB package (Brooks et al. 2017). We calculated and set the average relative growth rate of each individual (log[change in plant size(cm^2^)]/week; Gibson 2002, p. 214) as the response variable. We included the week of re‐emergence, disturbance treatment, and their interaction as fixed effects. We tested for multicollinearity between re‐emergence and disturbance treatments by calculating variance inflation factors to ensure that the effects on growth rate were independent from each other. We also included snow treatment as a fixed effect. We included a random effect for species in the model to control for the confounding influence of interspecific differences in growth rate. Finally, we included a random effect for subplot within plot within block to account for repeated measures. Because we included their interaction in the model, we evaluated estimated marginal means of disturbance treatments conditionally on emergence (~Fire | Emergence). Snow treatments did not significantly affect growth rate, so we conducted post hoc pairwise comparisons focused only on the effects of fire and week of re‐emergence.

To test H2b, whether re‐emergence timing, growth rate, or treatments influenced flowering effort, we used a binomial generalized linear mixed‐effects model via glmmTMB (Brooks et al. 2017). We set whether an individual flowered or not as the binary response variable and re‐emergence week, average relative growth rate, disturbance treatment, and snow treatment as fixed effects. We tested for multicollinearity between fixed effects by calculating the variance inflation factors to ensure that their effects on flowering were independent from each other. We again set species and subplot within plot within block as random effects.

We also used generalized linear mixed‐effects models to examine the number of flowering individuals and proportion of flowering individuals out of the total number of individuals in each disturbance and snow treatment. We set the number of flowering individuals and the proportion of flowering individuals as the response variable in their respective models. For the model of total number of flowering individuals, we set disturbance treatment and snow treatments as fixed effects. For the model of the proportion of individuals that flowered, we set disturbance treatment, snow treatment, and their interaction as fixed effects. In both models, we set subplot within plot within block as a random effect.

## RESULTS

### Re‐emergence timing and number of individuals

**FIGURE 3:**
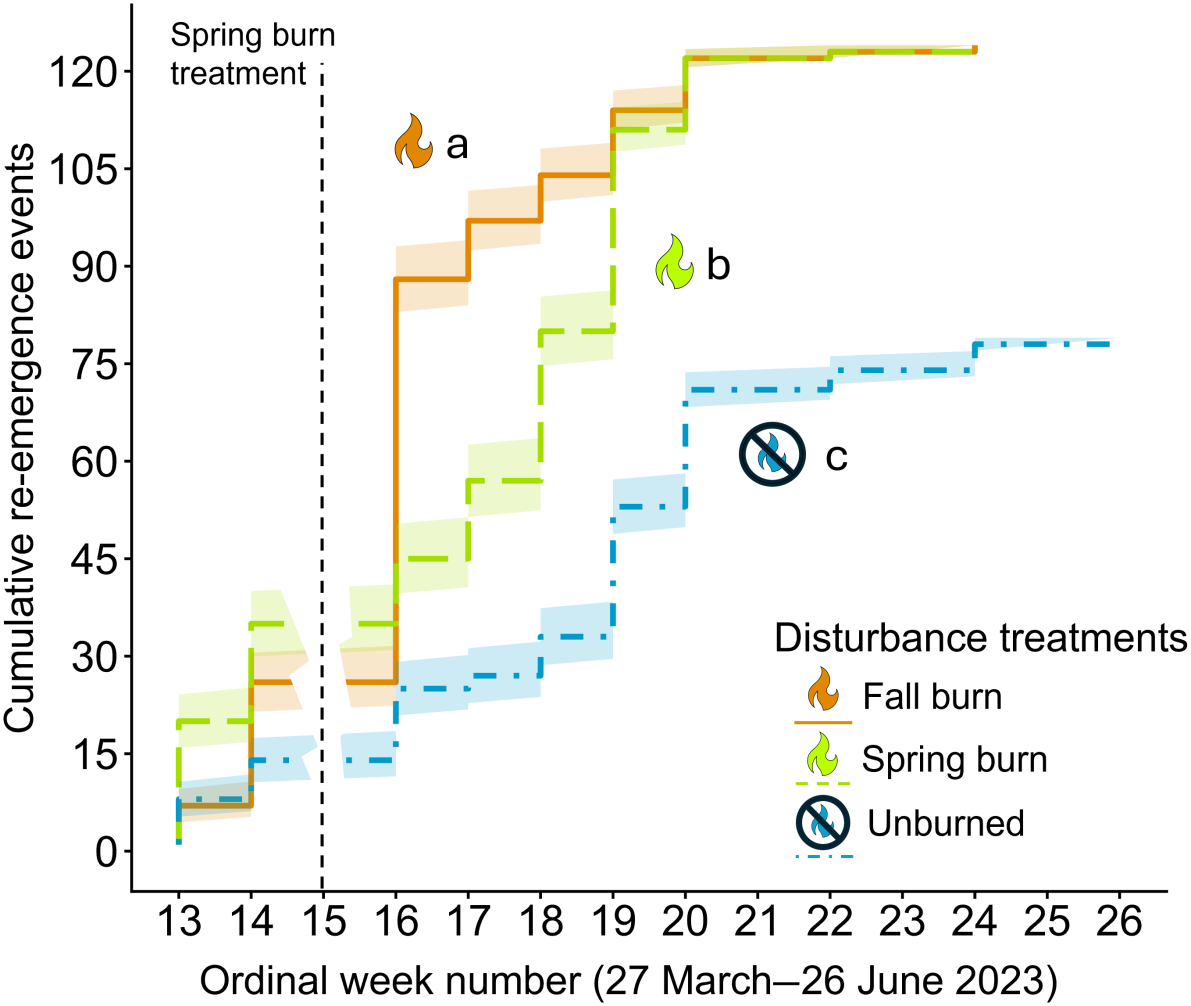
Re‐emergence happened significantly faster in fall burn treatments when compared to spring burn and unburned treatments as well as in spring burn treatments compared to unburned treatments. Rate of re‐emergence did not differ between snow treatments within the same fire treatment, so only differences between fire treatments are shown here, for clarity. Ordinal week numbers correspond to the week of 3 April through the week of 26 June 2023. No data were collected in Week 15, the week during which the spring prescribed burn was conducted on 18 April 2023. Shading indicates one SE. *n* = 124 for both spring burn and fall burn treatments and *n* = 79 for the unburned treatment. Differences in emergence timing were determined using pairwise estimated marginal means with a Holm adjustment for multiple comparisons. Figure created by Michelle Homann using icons of fire from PowerPoint.

Over the course of the growing season, the instantaneous rate of emergence was significantly greater in fall burn treatments than in spring burn or unburned treatments (*z* = 2.951, *p* = 0.0032 and *z* = 5.989, *p* < 0.0001, respectively). Re‐emergence rate was also greater in spring burn treatments than in unburned treatments (*z* = 3.672, *p* = 0.0005; Figure 3). There were significantly more individuals observed in fall and spring burn treatments than in unburned treatments throughout the growing season (*t*(39) = 2.979, *p* = 0.0149 and *t*(39) = 2.440, *p* = 0.0387, respectively). The total number of individuals that re‐emerged in fall and spring burn treatments was equal at the end of the growing season (*n* = 124), while there were approximately 36% fewer individuals in the unburned treatments (*n* = 79). Snow treatments did not affect re‐emergence timing or the number of individuals that were present (*z* = 0.213, *p =* 0.8317 and *z* = −0.416, *p =* 0.6796, respectively).

### Growth rate

**FIGURE 4:**
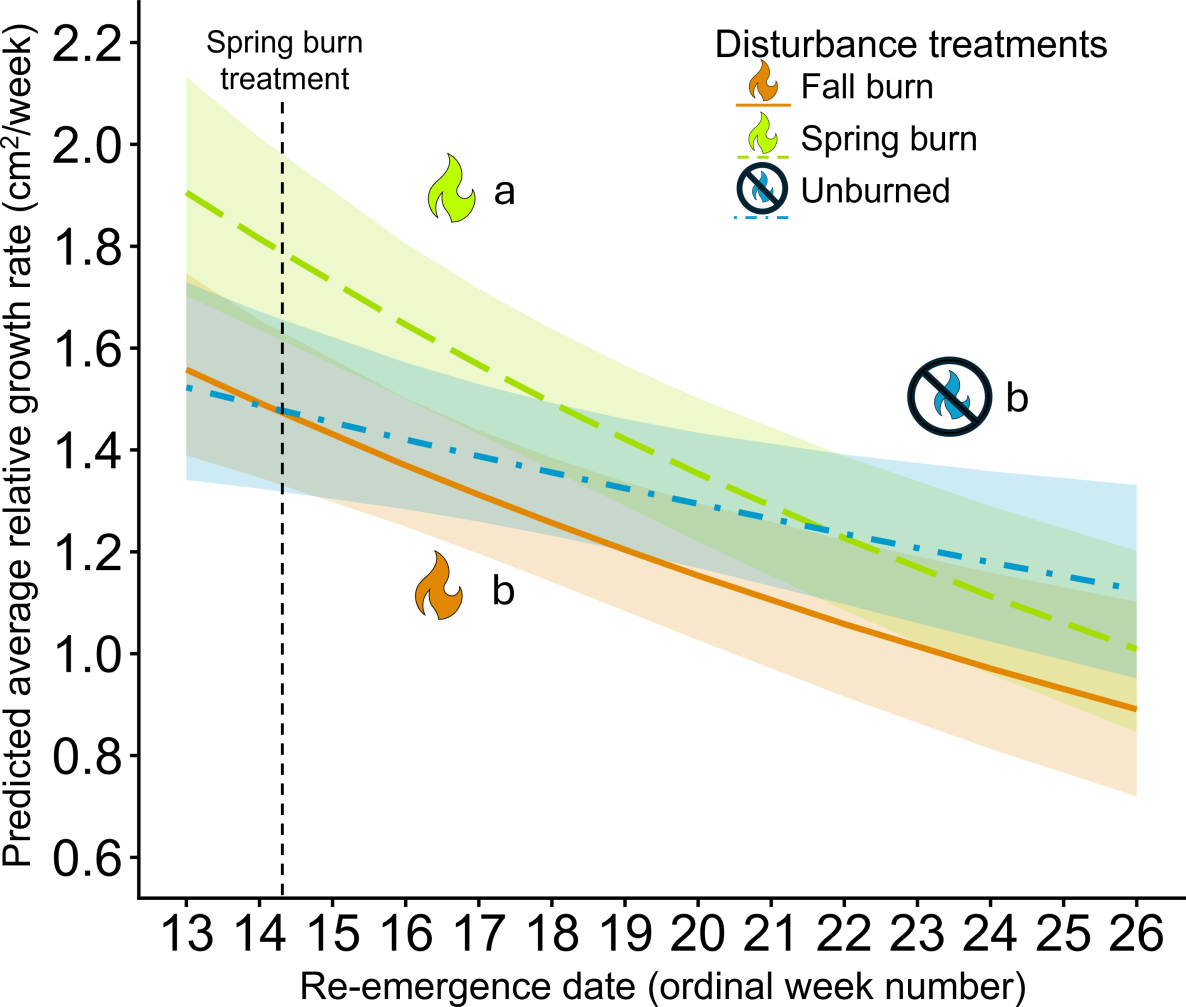
Average relative growth rate was higher for plants in spring burn treatments when compared to unburned and fall burn treatments. Predicted values for relative growth rate were modeled as a function of re‐emergence timing and disturbance treatment. Growth rate did not differ between snow treatments within the same fire treatment, so, for clarity, only differences between fire treatments are shown here. Ordinal week numbers correspond to the week of 3 April through the week of 26 June 2023. No data were collected in Week 15, during which the spring prescribed burn was conducted on 18 April 2023. The shaded areas indicate 95% CIs. Differences in relative growth rate were determined using pairwise estimated marginal means with a Holm adjustment for multiple comparisons. Figure created by Michelle Homann using icons of fire from PowerPoint.

Growth rate was negatively correlated with re‐emergence timing (*z* = −4.213, *p* < 0.0001), indicating that plants that re‐emerged early in the growing season grew faster than plants that re‐emerged later (Figure 4). Growth rate also differed between disturbance treatments, but patterns depended on the timing of re‐emergence. Growth rate was greater in spring burn treatments than in unburned and fall burn treatments for plants that re‐emerged between weeks 13 and 19 and between weeks 13 and 20, respectively (Table 2; Appendix S1: Figure S2). Growth rate was also greater in unburned treatments compared to fall burn treatments for plants that emerged between weeks 18 and 20 (*p* < 0.05; Table 2; Appendix S1: Figure S2). Snow treatments did not affect growth rate (*t*(305) = 0.526, *p* = 0.5995).

**TABLE 2:** Significant pairwise linear contrasts for individual growth rate depending on re‐emergence week and disturbance treatment with a Holm correction for multiple comparisons.

Re‐emergence week	Contrast	df	*t*‐ratio	*p*
13	Fall burn–Spring burn	305	−3.960	0.0003
13	Spring burn–Unburned	305	3.767	0.0004
14	Fall burn–Spring burn	305	−4.769	<0.0001
14	Spring burn–Unburned	305	3.960	0.0002
16	Fall burn–Spring burn	305	−6.781	<0.0001
16	Spring burn–Unburned	305	4.195	0.0001
17	Fall burn–Spring burn	305	−6.742	<0.0001
17	Spring burn–Unburned	305	3.885	0.0003
18	Fall burn–Spring burn	305	−5.584	<0.0001
18	Fall burn–Unburned	305	−2.199	0.0286
18	Spring burn–Unburned	305	3.056	0.0049
19	Fall burn–Spring burn	305	−4.298	0.0001
19	Fall burn–Unburned	305	−2.336	0.0402
19	Spring burn–Unburned	305	1.987	0.0478
20	Fall burn–Spring burn	305	−3.318	0.0030
20	Fall burn–Unburned	305	−2.317	0.0423

### Reproductive responses

**FIGURE 5:**
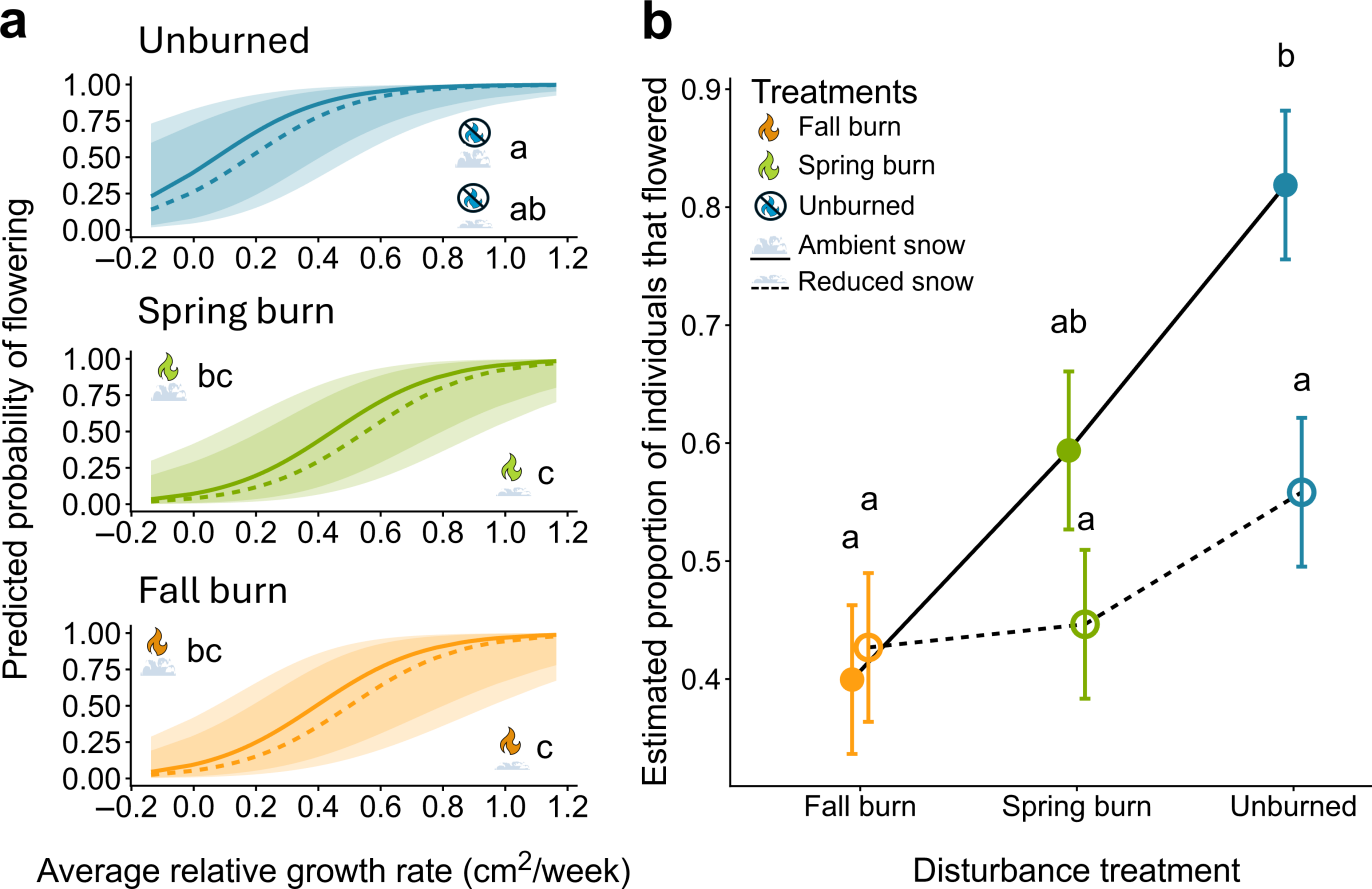
Average relative growth rate was correlated with an increased likelihood of flowering regardless of fire and snow treatments, and probability of flowering was generally higher for plants in unburned treatments than in either burn treatment (a). The model‐estimated proportion of flowering individuals was higher in unburned treatments with ambient snow than in all other treatments except spring burns with ambient snow (b). Shaded areas indicate 95% CIs (a), and lower case letters indicate significant differences in the likelihood of flowering (a) and proportion of flowering individuals (b). Significance was determined using pairwise estimated marginal means with a Holm adjustment for multiple comparisons. Figure created by Michelle Homann using icons of fire and snow from PowerPoint.

Emergence timing did not significantly affect flowering probability (*z* = −0.668, *p* = 0.5040), but relative growth rate had a significant positive effect on flowering probability (*z* = 3.797, *p* = 0.0001). Additionally, flowering probability was significantly higher in unburned treatments with ambient snow compared to fall burn and spring burn treatments regardless of snow treatment (Table 3; Figure 5a). Likewise, flowering probability was higher in no burn treatments with reduced snow than in fall and spring burn treatments with reduced snow (*z* = 2.855, *p* = 0.0473 and *z* = 3.190, *p* = 0.0199, respectively; Table 3; Figure 5a).

**TABLE 3:** Pairwise linear contrasts for the probability of flowering depending on disturbance and snow treatments with a Holm correction for multiple comparisons.

Contrast	*z*‐ratio	*p*
Unburned, ambient snow–Fall burn, ambient snow	2.855	**0.0473**
Unburned, ambient snow–Spring burn, ambient snow	3.190	**0.0199**
Unburned, ambient snow–Unburned, reduced snow	1.527	0.8865
Unburned, ambient snow–Fall burn, reduced snow	3.036	**0.0288**
Unburned, ambient snow–Spring burn, reduced snow	3.302	**0.0144**
Fall burn, ambient snow–Spring burn, ambient snow	0.498	1.0000
Fall burn, ambient snow–Unburned, reduced snow	−1.728	0.6719
Fall burn, ambient snow–Fall burn, reduced snow	1.527	0.8865
Fall burn, ambient snow–Spring burn, reduced snow	1.255	0.8865
Spring burn, ambient snow–Unburned, reduced snow	−2.096	0.3249
Spring burn, ambient snow–Fall burn, reduced snow	0.438	1.0000
Spring burn, ambient snow–Spring burn, reduced snow	1.527	0.8865
Unburned, reduced snow–Fall burn, reduced snow	2.855	**0.0473**
Unburned, reduced snow–Spring burn, reduced snow	3.190	**0.0199**
Fall burn, reduced snow–Spring burn, reduced snow	0.498	1.0000

*Note*: Bold text indicates significant pairwise linear contrasts.

The number of individuals that flowered within each treatment was statistically similar (*p* > 0.05), but the proportion of flowering individuals out of the total number of individuals within each treatment was higher in unburned treatments with ambient snow than in all other treatment combinations except spring burns with ambient snow (Table 4; Figure 5b). There was also significant interaction between fire and snow treatments (χ^2^ = 7.7883, *p* = 0.02036).

**TABLE 4:** Pairwise linear contrasts for the proportion of flowering individuals depending on disturbance and snow treatments with a Holm correction for multiple comparisons.

Contrast	df	*t*‐ratio	*p*
Unburned, ambient snow–Fall burn, ambient snow	37	4.701	**0.0005**
Unburned, ambient snow–Spring burn, ambient snow	37	2.446	0.2128
Unburned, ambient snow–Unburned, reduced snow	37	3.548	**0.0129**
Unburned, ambient snow–Fall burn, reduced snow	37	4.395	**0.0013**
Unburned, ambient snow–Spring burn, reduced snow	37	4.175	**0.0023**
Fall burn, ambient snow–Spring burn, ambient snow	37	−2.111	0.4156
Fall burn, ambient snow–Unburned, reduced snow	37	−1.781	0.6207
Fall burn, ambient snow–Fall burn, reduced snow	37	−0.371	1.0000
Fall burn, ambient snow–Spring burn, reduced snow	37	−0.526	1.0000
Spring burn, ambient snow–Unburned, reduced snow	37	0.385	1.0000
Spring burn, ambient snow–Fall burn, reduced snow	37	1.815	0.6207
Spring burn, ambient snow–Spring burn, reduced snow	37	1.919	0.5650
Unburned, reduced snow–Fall burn, reduced snow	37	1.475	0.8917
Unburned, reduced snow–Spring burn, reduced snow	37	1.255	1.0000
Fall burn, reduced snow–Spring burn, reduced snow	37	−0.220	1.0000

*Note*: Bold text indicates significant pairwise linear contrasts.

## DISCUSSION

Examining re‐emergence patterns and subsequent life‐history events in response to disturbance and climate change is necessary to build a comprehensive understanding of restoration outcomes in perennial plant communities (Brudvig 2017; Ott et al. 2019). Here, we assess the effects of fire application and timing on re‐emergence, growth, and flowering in perennial tallgrass prairie species and demonstrate cascading relationships between re‐emergence and growth rate and between growth rate and flowering. Similar to other studies regarding the effects of winter climate change in tallgrass prairies (e.g., Charton et al. 2025, Henn & Damschen 2022), the variables we measured showed limited responses to reduced snow despite 7 years of repeated snow manipulations.

Fall burn treatments had both lower litter depth (Appendix S1: Figure S3) and warmer temperatures early in the growing season when compared to other treatments (Appendix S1: Figure S4), supporting that reduced physical impedance from litter and warmer temperatures can expedite re‐emergence (Lubbe & Henry 2019, Livensperger et al. 2016). Despite similar litter depth between unburned and spring burn treatments prior to the spring burn (Appendix S1: Figure S3), litter had been accumulating for 7 years in the unburned treatments, as opposed to one growing season in the spring burn treatments. Faster emergence in spring burn treatments than in unburned treatments, while not a response we were expecting, further suggests that litter accumulation is an important factor in perennial plant re‐emergence dynamics. In addition to slower emergence, we also observed fewer individuals in unburned treatments, indicating that litter accumulation and lack of disturbance can have negative effects on disturbance‐adapted plants (Carson and Peterson 1990, Alstad et al. 2016).

Individuals that re‐emerged early in the growing season were able to grow quickly by capitalizing on nutrients and available space, while plants that re‐emerged later grew more slowly (Körner et al. 2008). The slope of the relationship between re‐emergence and growth did not differ between spring and fall burn treatments (Figure 4); instead, plants in spring burn treatments grew consistently faster across the range of re‐emergence dates. A combination of insulative litter to retain heat and moisture through the early growing season (Deutsch et al. 2010) and the removal of litter creating space and allowing more sunlight to reach the soil surface could contribute to the higher growth rate we observed in spring burn treatments (Liu et al. 2022). Growth rate in unburned treatments was intermediate, indicating a trade‐off between the insulative and impeding properties of accumulated litter (Lubbe and Henry 2019). Additionally, plants in fall burn treatments were subjected to colder and more variable winter conditions (Henn & Damschen 2022), including more freeze–thaw cycles than plants in other treatments (Appendix S1: Figure S5). Damage from frost can reduce growth (Lubbe & Henry 2019), but the minimum temperatures we observed through winter prior to data collection were likely not low enough to damage plant tissue, even in the coldest treatments (Appendix S1: Figure S4; Ladwig et al. 2018). Lack of insulation across 7 years of fall burn treatments prior to this study, however, could lead to the slower growth rate in fall burn treatments by driving an adaptive trade‐off between cold tolerance and growth strategy (Ding et al. 2019; Koehler et al. 2012; Ladwig et al. 2018; Savage & Cavender‐Bares 2013).

Similar to other studies regarding connections between growth strategies and flowering (Dybing & Grady 1994; Giménez et al. 2013; Méndez‐Vigo et al. 2010), we observed a link between individual growth rate and flowering probability (Figure 5a). A greater probability of flowering in unburned treatments when compared to disturbance treatments (Figure 5a) was somewhat surprising, as burning is generally understood to bolster flowering for many tallgrass prairie species (Hulbert 1988; Mola & Williams 2018; Richardson & Wagenius 2022). A higher proportion of flowering individuals in the unburned treatment with ambient snow could be a response to the soil moisture input from ambient snow in combination with moisture retention and nutrient input from years of litter accumulation and decomposition at the soil surface (Zhong et al. 2017). Additionally, burn treatments were applied annually in the 7 years leading up to this experiment, which is more frequent than the historical 3‐ to 5‐year fire return interval in tallgrass prairies (Allen & Palmer 2011). These frequent disturbances may have driven a trade‐off between vegetative resprouting and maintenance versus floral reproduction (Clarke & Dorji 2008). A drought in the summer of 2023 may have further limited flowering disproportionately in the burn treatments, which lacked litter to retain moisture when compared to the unburned treatments (Deutsch et al. 2010; Dietrich & Smith 2016).

We are hesitant, however, to imply that a higher probability of flowering in unburned treatments indicates a benefit to fire suppression. We observed fewer individuals in unburned treatments, while the number of individuals that flowered was statistically similar across treatments (Table 5). This difference in the proportion of flowering individuals out of the total number of individuals observed contributed to a higher probability of flowering in unburned treatments despite similar floral abundance across treatments (Figure 5b). Further, observing fewer individuals in unburned treatments reinforces the importance of fire to restore and maintain plant populations in prairies by reducing litter accumulation (Letts et al. 2015). Accumulation of litter generally decreases seedling recruitment and establishment in this system (Anderegg et al. 2022; Ruprecht et al. 2010), so this difference in the proportion of sexually mature individuals in unburned treatments could be driven by a lack of recruitment in recent years.

**TABLE 5:** Total number of individuals present at the end of the growing season and proportion of individuals that flowered.

Fire treatment	No. individuals	No. flowering individuals	Proportion flowering
Unburned	63	40	0.63
Fall burn	91	37	0.40
Spring burn	81	41	0.51

While we found strong relationships between the timing of fire and life‐history events of perennial prairie plants, only flowering dynamics responded to winter climate treatments. Climate is variable, and we only had one snowfall event exceed the 10‐cm threshold at which snow removal treatments would be applied in winter prior to this study (Appendix S1: Table S1). Despite the difficulty of maintaining a control treatment in reference to projected future climate conditions when the climate is actively changing (Lubbe & Henry 2020), soil temperature and moisture data from a subsequent field season suggest that even a single snow manipulation treatment can have lasting effects on soil temperature and moisture through winter and into spring (Homann & Damschen, unpublished manuscript). Given the real effects of snow manipulations on microclimate conditions, in combination with several years of snow manipulations leading up to this study, we cautiously conclude that a lack of response indicates resilience to reduced snow cover, at least in the short term (Chandler & Travers 2022; Charton et al. 2025; Henn & Damschen 2022). Limited detectable responses to climate change in long‐lived perennial systems in the short term, however, do not preclude plant community responses to climate change across longer timescales. For example, the trend of reduced snow treatments reducing flowering probability within respective disturbance treatments, though not significant, could be an early indicator of responses that begin to matter over time (Figure 5a). Temperate grasslands are expected to be sensitive to future climate shifts (Li et al. 2018, Lubbe & Henry 2020), and rare species may respond differently than the well‐represented species we focused on in this study (Leach & Givnish 1996).

### Conclusion and management implications

While studies of plant community change often focus broadly on diversity metrics, here we examined early growing season dynamics as a proximal response to disturbance. We observed phenological and fitness responses to fire timing and snow treatments that were not detected by diversity or richness metrics measured during previous research within this long‐term experiment (Charton et al. 2025; Henn & Damschen 2022). Despite the uncertainty that climate change brings to grassland management, our results suggest resilience to climate change in the short term. Management should focus on varying dormant‐season burn timing between spring and fall, as the effect of fire differs seasonally. Fall prescribed fire could promote resilience in plant communities as winter conditions become more variable (Ladwig et al. 2018), while spring prescribed fire maintains warmer temperatures by leaving litter intact over winter into early spring. We note that burning annually in the same season likely hinders the reproductive benefits of fire that could be realized across longer fire return intervals and suggest a fire regime more similar to a natural fire history (e.g., 2–3 years; Allen & Palmer 2011). With uncertainty comes the need to adapt; conducting prescribed burns in winter or early spring before native species begin to resprout is becoming more feasible with warmer winters. Future research should explore a range of dormant‐season burn timing between fall and spring, which may help strike a balance between maintaining insulation during the coldest part of the year and reducing the likelihood of damage to actively growing plants in spring.

## AUTHOR CONTRIBUTIONS

Both authors contributed to idea generation and study design. Michelle A. Homann led data collection and curation, visualization, formal analysis, and writing of the initial manuscript draft. Ellen I. Damschen contributed to manuscript editing and review and provided lab infrastructure and supervision. Both authors are researchers at a US‐based university and have interests in the conservation, restoration, and management of fire‐adapted grasslands. The authorship team represents the region of study, as we live, work, and recreate in the area, contributing to appropriate interpretation of results. Our research was discussed with local managers to seek feedback on questions, methods, and interpretation.

## CONFLICT OF INTEREST STATEMENT

The authors declare no conflicts of interest.

## Supporting information


Appendix S1.

## Data Availability

Data (Homann, 2026) are available on ScienceBase at https://doi.org/10.5066/P13C4KDN.
