# Supplementary material for: Effects of fire timing and snow cover on tallgrass prairie plant re‐emergence phenology, growth rate, and flowering
Source: Ecol Appl. 2026 Apr 9;36(3):e70213. doi: 10.1002/eap.70213 (PMC13063366; doi:10.1002/eap.70213)
Supplement: Supplementary file 1 — Appendix S1. [file EAP-36-e70213-s001.pdf]

APPENDIX S1

# Effects of fire timing and snow cover on tallgrass prairie plant re-emergence phenology, growth rate, and flowering

Michelle A. Homann, Ellen I. Damschen

*Ecological Applications*

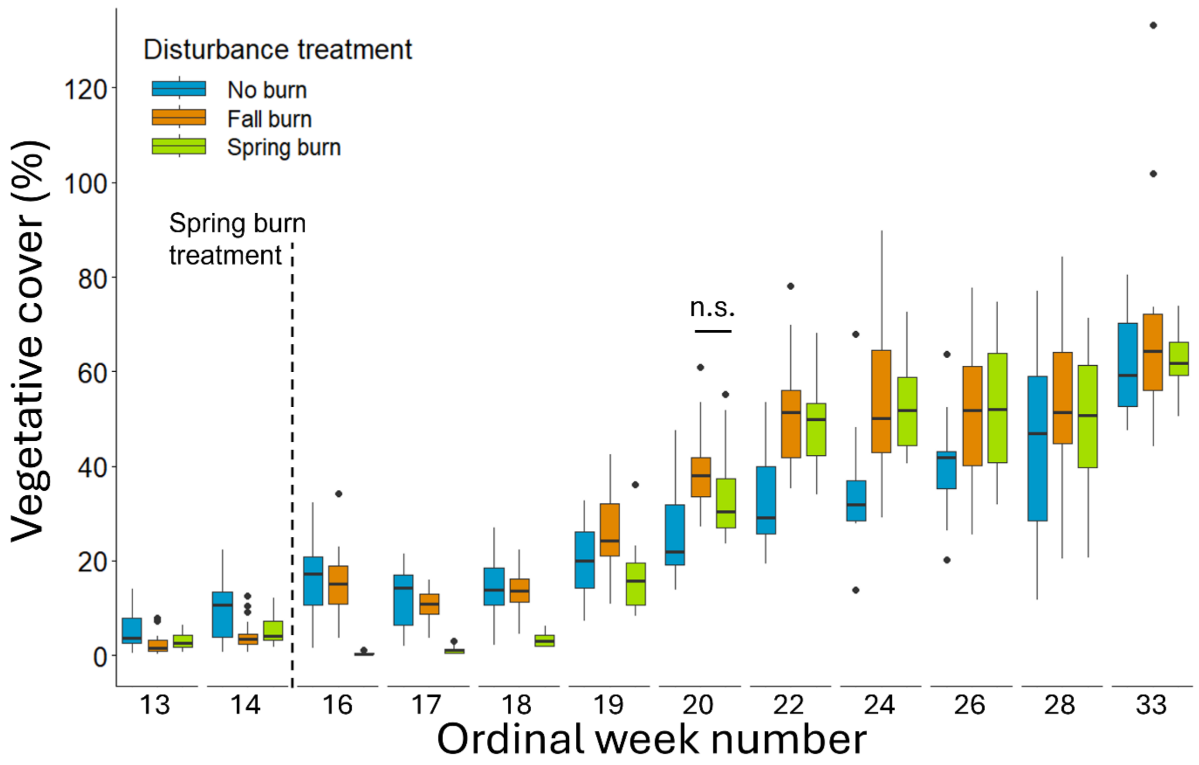


FIGURE S1. Percent vegetative cover no longer differed between spring and fall burn treatments by ordinal week 20. Lower-case letters indicate significance at α = 0.05.

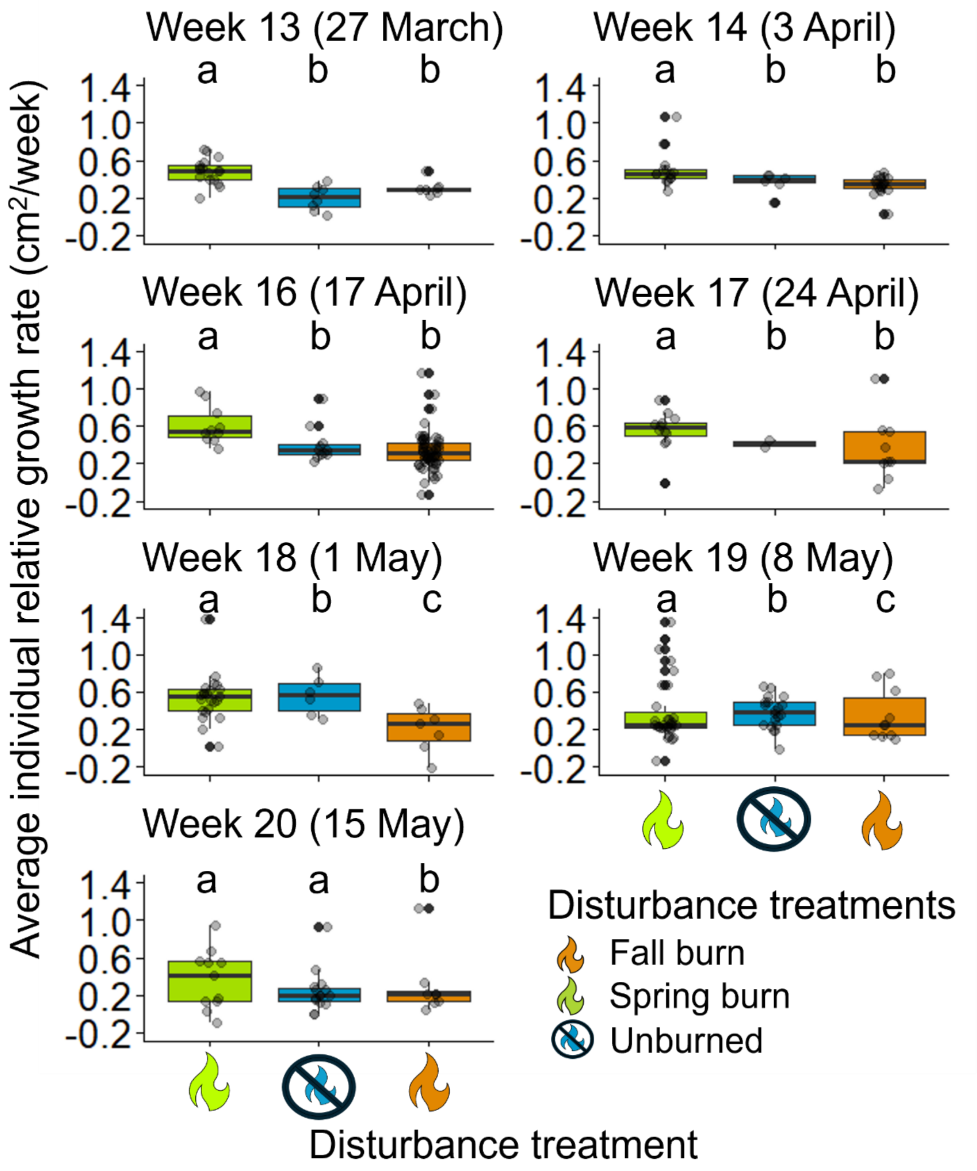


Figure S2. Growth rate was greater in spring burns than in fall burn and unburned treatments from the week of 27 March through 8 May 2023 and remained greater than fall burn treatments throughout the growing season. Growth rate was greater in unburned treatments than in fall burn treatments from the week of 1 May through 15 May 2023. Lower-case letters indicate significant differences at differences at α = 0.05. Figure created by Michelle Homann using icons of fire from PowerPoint.

LITTER AND SOIL RESPONSES TO DISTUBRANCE AND SNOW TREATMENTS

To validate the effect of prescribed fire timing on ground cover and soil temperature, we measured litter depth in the four corners of each subplot and visually estimated the percent of each subplot that was covered by litter during the weeks of 26 March, 2 April, and 16 April 2023. We calculated an area-weighted litter depth (cm) by multiplying mean litter depth in each subplot by the percent of the subplot area covered by litter. Additionally, we placed iButton data loggers (DS1921G-F5# Thermochron, 4K, iButtonLinkTechnology) 2 cm below the soil surface in the center of each subplot to record soil temperature every two hours from November through June 2023.

To evaluate the effects of disturbance and snow depth on litter depth and soil temperature, we used generalized linear mixed effects models with log-litter depth and average monthly temperature as response variables using the glmmTMB package (Brooks et al. 2017). We evaluated log-litter depth to meet normality assumptions in the litter depth models. We included disturbance treatment and snow treatment as fixed effects in each model. We also included a random effect for subplot within plot within block.

Prior to the spring burn, litter depth was significantly lower in fall burn treatments than in spring and unburned treatments regardless of snow treatment ($t(40) = -19.983$, $p < 0.0001$, $t(40) = -20.639$, $p < 0.0001$, respectively). Litter depth no longer differed between fall and spring burn treatments after the spring burn was conducted ($t(40) = 0.037$, $p = 0.9705$), at which point unburned treatments had greater litter depth than both fall and spring burn treatments ($t(40) = 13.050$, $p < 0.0001$, $t(40) = 13.087$, $p < 0.0001$, respectively).

Trends in mean soil temperature varied depending on the time of year. Soil temperatures in unburned treatments trended highest during winter months (December-February) and lowest spring (March-May) when compared to fall and spring burn treatments. Mean temperatures in spring burn treatments were typically intermediate between fall and unburned treatments until after the spring burn, at which point we observed significantly higher minimum temperatures in spring burn treatments than in other treatments.

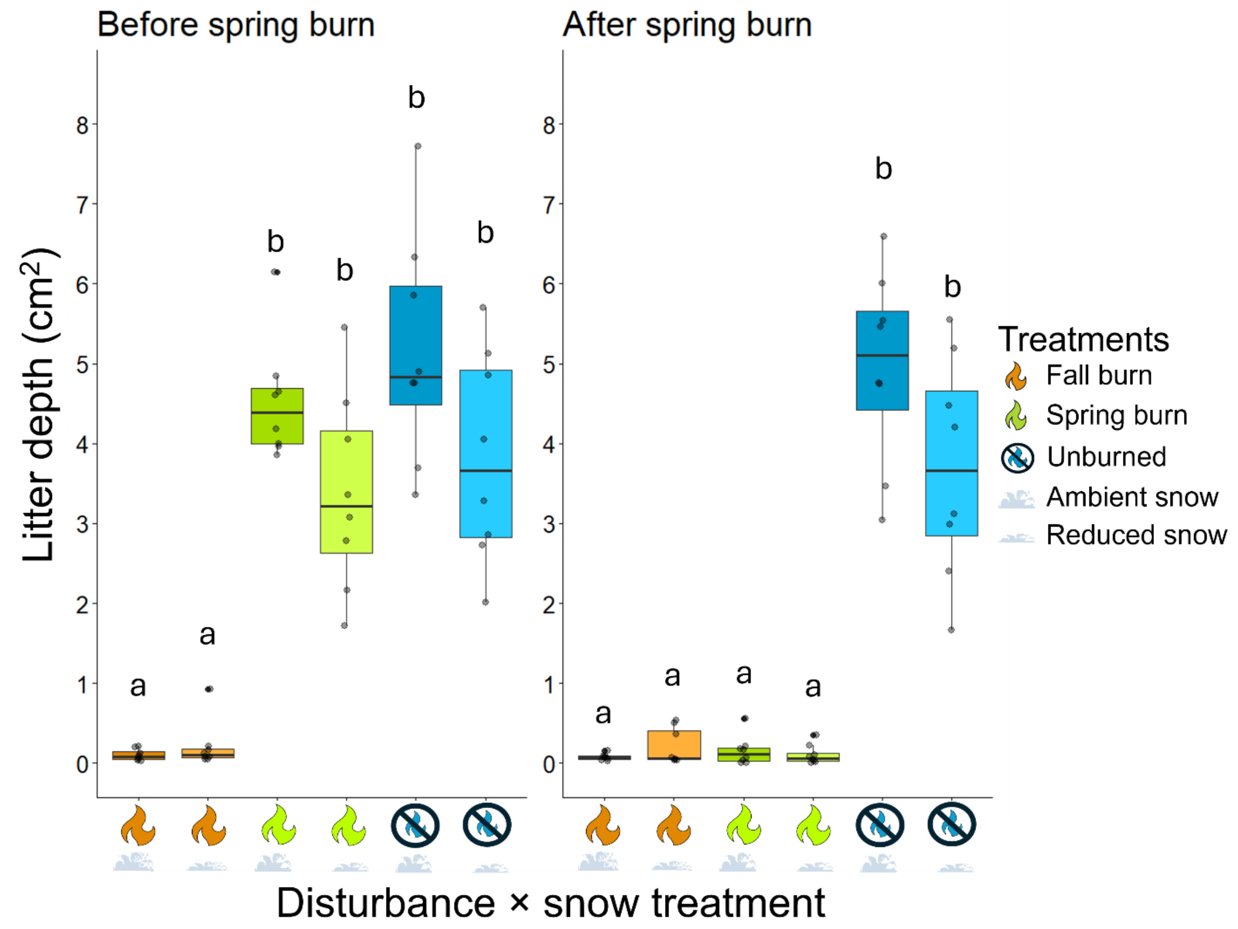


Figure S3. Prior to the spring burn, litter depth was higher in spring burn and unburned treatments than in fall burn treatments. Following the spring burn, litter depth no longer differed between spring and fall burn treatments, both of which had lower litter depth than in the unburned treatment. Lowercase letters indicate significant differences at α = 0.05. Figure created by Michelle Homann using icons of fire and snow from PowerPoint.

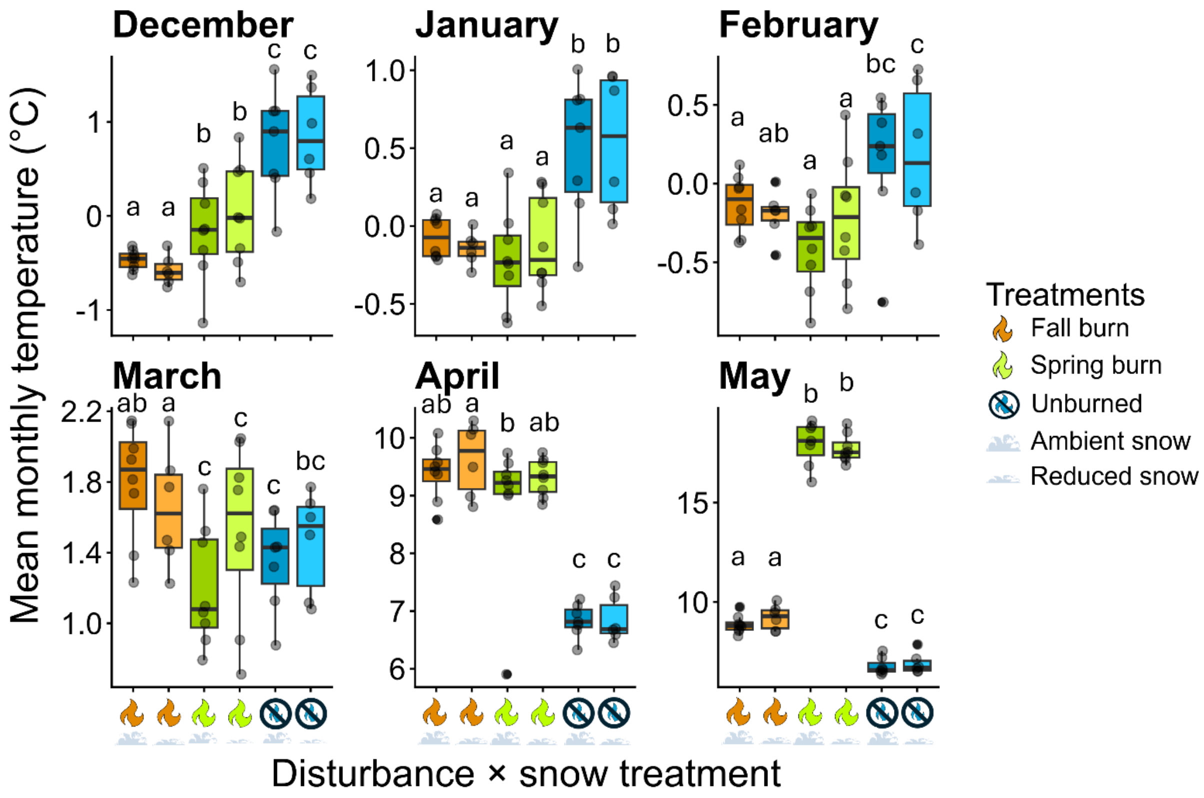


Figure S4. Soil temperature trended higher in unburned treatments than in burned treatments in winter months and lower in unburned treatments than in burned treatments in spring months. Lowercase letters indicate significant differences at $\alpha = 0.05$. Figure created by Michelle Homann using icons of fire and snow from PowerPoint.

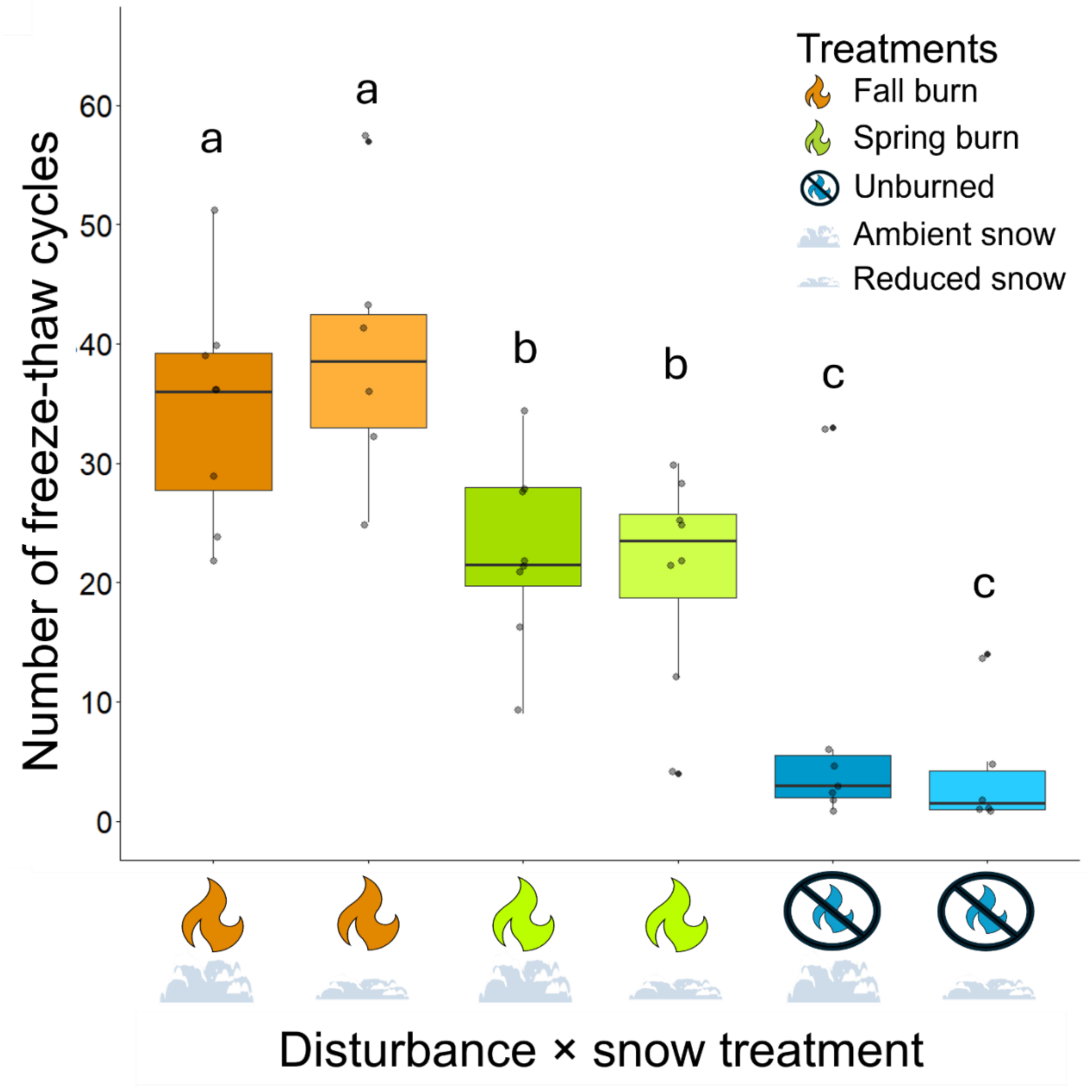


Figure S5. More freeze-thaw cycles occurred in fall burn treatments than in spring burn or unburned treatments and in spring burn treatments than in unburned treatments. Lowercase letters indicate significant differences at α = 0.05. Figure created by Michelle Homann using icons of fire and snow from PowerPoint.

TABLE S1. Number of snow manipulations in each of seven years leading up to data collection.

| Winter season | 2016 - 2017 | 2017 - 2018 | 2018 - 2019 | 2019 - 2020 | 2020 - 2021 | 2021 - 2022 | 2022 - 2023 |
|---|---|---|---|---|---|---|---|
| Snow manipulations | 3 | 3 | 6 | 0* | 4 | 2 | 1 |

*Snow manipulations were not conducted due to COVID-19 work restrictions.
